# Disruption of GPR35 Signaling in Bone Marrow-Derived Cells Does Not Influence Vascular Inflammation and Atherosclerosis in Hyperlipidemic Mice

**DOI:** 10.3390/metabo11070411

**Published:** 2021-06-23

**Authors:** Roland Baumgartner, Felipe B. Casagrande, Randi B. Mikkelsen, Martin Berg, Konstantinos A. Polyzos, Maria J. Forteza, Aastha Arora, Thue W. Schwartz, Siv A. Hjorth, Daniel F. J. Ketelhuth

**Affiliations:** 1 Division of Cardiovascular Medicine, Center for Molecular Medicine, Department of Medicine, Karolinska Institute, Karolinska University Hospital, 17164 Stockholm, Sweden; roland.baumgartner@ki.se (R.B.); felipe.casagrande@ki.se (F.B.C.); clmbbbk@gmail.com (M.B.); konstantin.polyzos@gmail.com (K.A.P.); maria.forteza.de.los.reyes@ki.se (M.J.F.); aastha.arora@ki.se (A.A.); 2 Novo Nordisk Foundation Center for Basic Metabolic Research, University of Copenhagen, 2200 Copenhagen N, Denmark; mikkelsen@sund.ku.dk (R.B.M.); tws@sund.ku.dk (T.W.S.); sivhjorth@sund.ku.dk (S.A.H.); 3 Department of Cardiovascular and Renal Research, University of Southern Denmark, J.B. Winsløws vej 21, 5000 Odense C, Denmark

**Keywords:** atherosclerosis, GPR35, inflammation, immunometabolism, kynurenine, tryptophan, macrophages

## Abstract

G-protein-coupled receptor-35 (GPR35) has been identified as a receptor for the tryptophan metabolite kynurenic acid (KynA) and suggested to modulate macrophage polarization in metabolic tissues. Whether GPR35 can influence vascular inflammation and atherosclerosis has however never been tested. Lethally irradiated *LdlrKO* mice were randomized to receive *GPR35KO* or wild type (WT) bone marrow transplants and fed a high cholesterol diet for eight weeks to develop atherosclerosis. GPR35KO and WT chimeric mice presented no difference in the size of atherosclerotic lesions in the aortic arch (2.37 ± 0.58% vs. 1.95 ± 0.46%, respectively) or in the aortic roots (14.77 ± 3.33% vs. 11.57 ± 2.49%, respectively). In line with these data, no changes in the percentage of VCAM-1+, IA^b^ + cells, and CD3+ T cells, as well as alpha smooth muscle cell actin expression, was observed between groups. Interestingly, the GPR35KO group presented a small but significant increase in CD68+ macrophage infiltration in the plaque. However, in vitro culture experiments using bone marrow-derived macrophages from both groups indicated that GPR35 plays no role in modulating the secretion of major inflammatory cytokines. Our study indicates that GPR35 expression does not play a direct role in macrophage activation, vascular inflammation, and the development of atherosclerosis.

## 1. Introduction

Cardiovascular diseases (CVDs), including myocardial infarction and stroke, are the leading cause of death worldwide [1]. The most common cause of CVDs is atherosclerosis, a chronic inflammatory disease affecting large- and medium-sized arteries. Atherosclerosis is initiated by the retention and accumulation of low-density lipoprotein (LDL) in the subendothelial space, triggering a maladaptive vascular and immune cell responses [2].

Novel insights into the processes driving inflammation have revealed that microenvironmental changes in metabolism can control immune cell functions. Hence, immunometabolism, which is how this field is recognized, has been implicated in several pathophysiological processes, including metabolic disorders, cancer, autoimmunity, as well as atherosclerosis [3].

For a very long time, metabolites have been considered as just ‘‘fuels’’, energy sources or building blocks, in cellular metabolic processes. However, recent research has changed this view and shown that many metabolites can also act as signaling molecules, for example interacting with metabolite sensing G-protein-coupled receptors (GPCRs) [4].

Approximately twenty metabolite-sensing GPCRs have been reported, including sensors for different length fatty acids (e.g., GPR41, GPR43, GPR84, GPR40, and GPR 120), lactate (GPR81), citric-acid cycle intermediates (e.g., GPR91 for succinate), and the amino acid tryptophan (GPR142), or one of its metabolites, kynurenic acid (GPR35) [4]. GPR35 expression is found in several tissues, as well as many immune cells, including monocytes/macrophages, granulocytes, dendritic cells, mast cells, and some T-cell subtypes [5,6,7]. In general, GPR35-mediated signaling has been implicated in promoting anti-inflammatory responses and to prevent disease, including colitis [8,9], mast cell-mediated allergic reactions [10], and adipose tissue inflammation in obesity [11]. Whether GPR35 plays a role in vascular inflammation and the development of atherosclerosis is unknown.

In the present study, we generated *LdlrKO* chimeric mice transplanted with *GPR35KO* or wild type (WT) bone marrow cells to investigate the role of this metabolite-sensing receptor in CVD. Despite previous evidence linking GPR35 with protective anti-inflammatory responses, GPR35KO and WT chimeric *LdlrKO* mice presented no detectable differences in the degree of vascular inflammation and atherosclerosis development. Further investigations using bone marrow-derived macrophages (BMDM) from *GPR35KO* and WT mice indicated that GPR35 plays no role in the activation of these cells, suggesting that any anti-inflammatory effects mediated by this receptor in vivo could be indirect and influenced by other mechanisms in different cells and tissues.

## 2. Results

### 2.1. GPR35KO and WT Chimeric Mice Show No Differences in Blood Parameters

**Figure 1:**
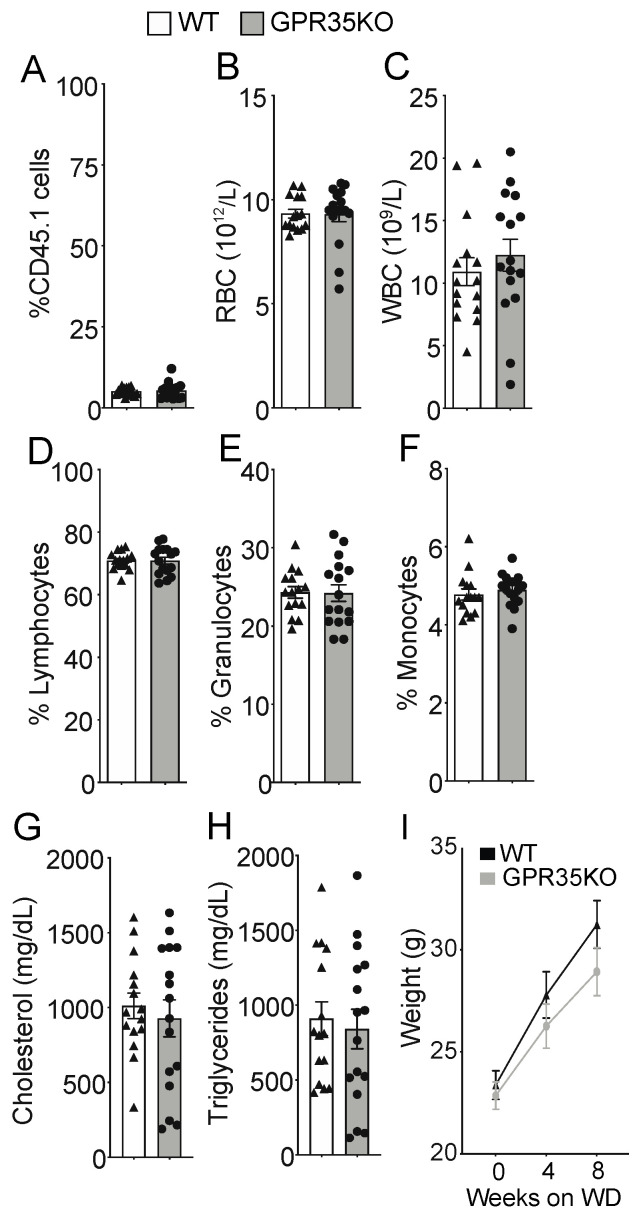
GPR35KO and WT *LdlrKO* chimeric mice present similar blood parameters and body weight. Thirteen week-old *LdlrKO* mice (expressing the CD45.1 allele) were irradiated and randomly assigned to receive CD45.2 allele expressing bone marrows from *GPR35KO* mice (*n* = 16; 8 females + 8 males) or WT mice (*n* = 15; 8 females + 7 males). After recovering for 4 weeks, mice were fed WD for 8 weeks. (**A**) Percentage of remaining host CD45.1+ cells in blood at the end of experiment (note: non-irradiated *LdlrKO* mice present 100% CD45.1+ cells). (**B**–**F**) Differential blood counts of (**B**) red blood cells (RBC), (**C**) white blood cells (WBC), (**D**) % of lymphocytes, (**E**) % of granulocytes, and (**F**) % of monocytes. (**G**) and (**H**) show plasma cholesterol and triglyceride levels. (**I**) Weight gain during the 8 weeks of WD feeding. Results are mean ± SEM. Pooled data from 2 independent experiments is shown. No significant differences were observed (**A**–**H**, Mann–Whitney; **I**, Two-way ANOVA).

*LdlrKO* mice, expressing the CD45.1 allele, were lethally irradiated and randomly assigned to receive either GPR35KO or WT bone marrow grafts, both expressing the CD45.2 allele. After eight weeks of western diet (WD) feeding, no difference was observed in the percentage of surviving host CD45.1+ cells (~5% in both groups; Figure 1A); these data indicate a chimerism >95% in both groups. Blood cell count analysis showed no difference in the number of red and white blood cells at the end of the experiment (Figure 1B,C). Hence, a similar percentage of lymphocytes, granulocytes, as well as monocytes was observed between the two groups (Figure 1D–F). Analysis of plasma samples also revealed no differences between groups for total cholesterol and triglyceride levels (Figure 1G,H). In line with the previous data, GPR35KO and WT chimeric mice presented no significant difference in weight gain during the eight weeks of western diet feeding (Figure 1I).

### 2.2. GPR35 Deficiency in Bone Marrow-Derived Cells Does Not Influence Atherosclerosis and Vascular Inflammation

**Figure 2:**
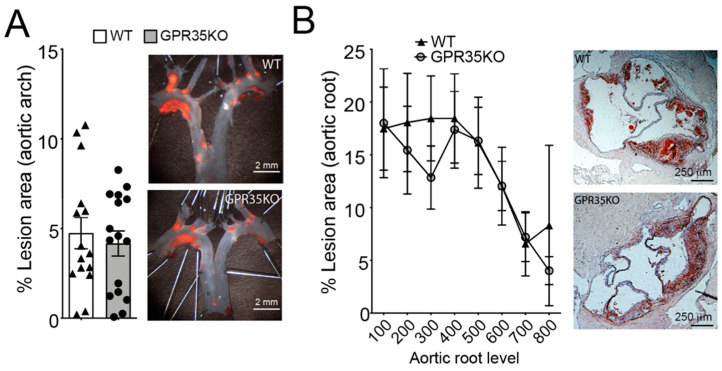
Ablation of GPR35 signaling in bone marrow-derived cells does not influence atherosclerosis. Thirteen week-old *LdlrKO* mice reconstituted with *GPR35KO*, and WT mice bone marrows were fed WD for 8 weeks. (**A**) Dissected aortic arches were stained with Sudan IV *en face* and % lesion areas of total vessel area are shown. Representative stained samples from each group are shown on right panels; *n* = 16 and 15, for GPR35KO and WT groups, respectively. Results are mean ± SEM. (**B**) Percentage atherosclerotic lesion area in the proximal aorta of male transplanted *LdlrKO;* *n* = 8 and 6, for GPR35KO and WT groups, respectively. Data show means of each of the 8 cross-sectional lesion sizes for the animals in the same group. Right panels show representative micrographs from each group. Pooled data from 2 independent experiments is shown. No significant differences were observed (**A**, Mann–Whitney; **B**, Two-way ANOVA).

*LdlrKO* mice transplanted with bone marrow cells from GPR35KO mice or WT littermate controls were used to assess the role of GPR35-deficient leukocytes in atherosclerosis. GPR35KO-reconstituted *LdlrKO* mice exhibited no differences in the percentage of aortic arch lesions compared to WT-reconstituted mice (Figure 2A). Similarly, no significant difference between groups was observed in the percentage of lesions developed in the aortic roots of chimeric mice, visualized by Oil Red O staining and quantitated by morphometric analysis (Figure 2B).

**Figure 3:**
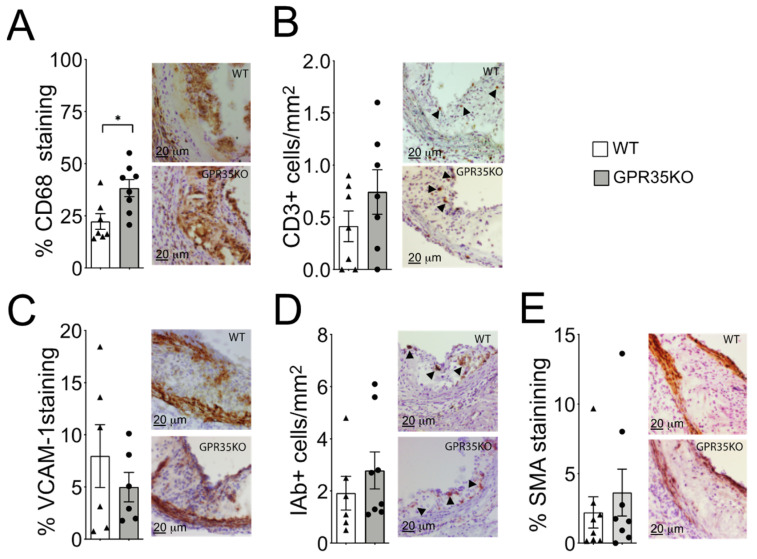
Effects of GPR35KO reconstitution on plaque inflammatory cell composition. Thirteen week-old *LdlrKO* mice reconstituted with *GPR35KO* or WT mice bone marrows were fed WD for 8 weeks. (**A**) Percentage of CD68+ macrophages, (**B**) number of CD3+ T cells/mm^2^, (**C**) percentage of VCAM-1+ stained cells, (**D**) the number of IA^b^+ cells/mm^2^, and (**E**) the percentage of α-smooth muscle cell actin (SMA) in both groups. Right panels show representative micrographs for each group. Results are mean ± SEM; *n* = 7–8. Mann–Whitney was used detect statistical differences. (*) *p* < 0.05.

Analysis of atherosclerotic plaque cell composition by immunohistochemistry staining of aortic roots revealed a small but significant increase in the percentage of infiltrating CD68+ macrophages in the GPR35KO group compared to WT group (Figure 3A). However, no significant differences in the number of CD3+ T cells, the percentage of adhesion molecule VCAM-1 expression, number of MHC-II IA^b^+ cells, or the percentage of α-smooth muscle actin were detected between the two groups (Figure 3B–E).

### 2.3. GPR35KO- and WT-Derived Bone Marrow-Derived Macrophages (BMDM) Respond Similarly in a Dose Response Manner to LPS Stimulation

**Figure 4:**
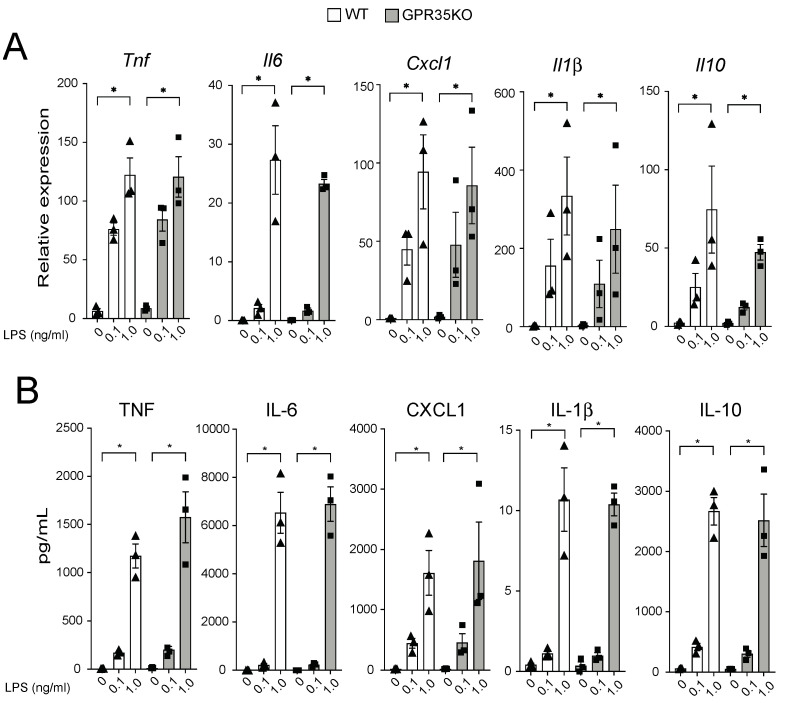
Dose-dependent response of GPR35KO and WT BMDMs to LPS. (**A**) Relative mRNA expression of *Tnf*, *Il6*, *Cxcl1*, *Il1β*, and *Il10* on BMDMs from GPR35KO and WT mice, after 2 h incubation with increasing doses of LPS; *n* = cells from 3 mice/ group. (**B**) Levels of TNF, IL-6, CXCL1, IL-1β, and IL-10 secreted by BMDMs from GPR35KO and WT mice, after 20 h incubation with increasing doses of LPS; *n* = cells from 3 mice/ group. Results are mean ± SEM. Friedman test with Dunn’s post-test was used for the pairwise comparisons. (*) *p* < 0.05.

The in vivo finding that the percentage of CD68+ macrophages was slightly, yet significantly, increased in the plaques from GPR35KO group prompted us to evaluate their response in vitro upon LPS stimulation. *GPR35KO* and WT BMDMs both promptly responded to LPS, in a very similar dose-dependent fashion, increasing mRNA levels of the pro-inflammatory genes *Tnf*, *Il6*, *Cxcl1*, and *Il1β*, and the anti-inflammatory gene *Il10* (Figure 4A). Similarly, LPS significantly increased the production of these same cytokines at the protein levels in the supernatant of cultures, and no differences between GPR35KO and WT groups were observed (Figure 4B). Altogether, this showed that the lack of GPR35 in bone marrow cells does not appear to influence macrophage activation through toll-like receptor stimulation.

### 2.4. GPR35 Signaling Does Not Influence Macrophage Responses to LPS

**Figure 5:**
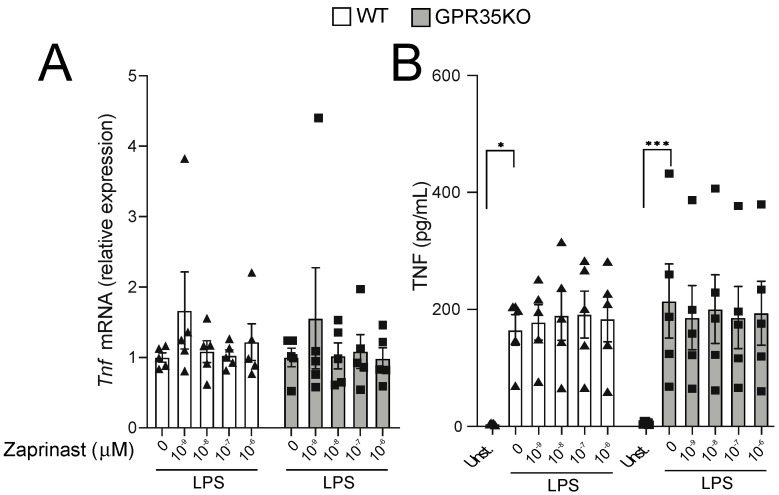
The effect of GPR35 ligation with zaprinast to TNF production on BMDMs stimulated with LPS. (**A**) The relative mRNA expression of *Tnf* in *GPR35KO* and WT BMDM, co-incubated for 2 h with increasing doses of zaprinast and 0.1 ng/mL LPS; *n* = cells from 5 mice/group. (**B**) Levels of TNF secreted by BMDMs from *GPR35KO* and WT mice, co-incubated for 2 h with increasing doses of zaprinast and 0.1 ng/mL LPS; *n* = cells from 5 mice/group. Results are mean ± SEM. No significant differences were observed; Friedman test with Dunn’s post-test. (*) *p* < 0.05, (***) *p* < 0.001.

Because the titration response to LPS was performed in the absence of GPR35 ligands in the well, we next conducted experiments in which GPR35KO and WT BMDM were stimulated with LPS, and in the presence of increasing concentrations of a GPR35 agonist. Using a receptor signaling assay with HEK 293 cells transfected with mouse GPR35, three distinct GPR35 agonist ligands were analyzed, including kynurenic acid, pamoic acid, and the synthetic agonist zaprinast. Based on this analysis, we chose zaprinast, the strongest GPR35 agonist, for our further macrophage studies in vitro (Figure S1). The latter experiment demonstrated that TNF mRNA transcripts and protein levels were not affected by increasing concentrations of zaprinast in neither GPR35KO nor WT BMDMs in combination with LPS stimulation (Figure 5A,B). A similar response was observed for the mRNA and protein levels of CXCL1 and IL-10 (Figure S2).

## 3. Discussion

Although GPR35 signaling has been proposed to trigger anti-inflammatory responses in the gut [8,9] and adipose tissue [11], we show that the ablation of GPR35 signaling in bone marrow-derived cells did not influence vascular inflammation and atherosclerosis in hyperlipidemic mice. While it is plausible, based on other studies, that GPR35 may in fact regulate inflammation, our data suggest that its major mechanism does not rely on direct down-stream effects on leukocytes.

Leukocyte infiltration into the arterial intima, in response to modified lipids and local immune activation, is considered as a key initiating step in the development of atherosclerosis. Notably, the pool of inflammatory cells in the plaque is known to also involve tissue-resident cells, established during embryonic development [12], as well as the processes of transdifferentiation of vascular cells [13]. In our study, reconstitution of *LdlrKO* mice with GPR35KO bone marrow did not affect hematopoiesis compared to controls, indicating that GPR35 signaling play no role in the development of major myeloid and lymphoid immune cell lineages, as well as erythrocytes. This goes in line with the fact that although GPR35 expression has been associated with certain pathological processes, particularly in the gastro-intestinal tract [14], it has to date not been associated with blood-related disorders.

A GWAS analysis study has identified an association between a single nucleotide polymorphism (rs3749172) in GPR35 and a high degree of coronary artery calcification (CAC) measured by computed tomography, which is used as a surrogate marker of the total burden of atherosclerosis [15]. Also in the context of CVDs, high GPR35 expression in the heart has been associated with heart failure [16]. Considering the previous links to inflammation here described, it was reasonable to hypothesize that GPR35 ablation would influence the process of atherogenesis. Surprisingly, our study showed that lack of GPR35 expression in circulating blood cells neither affected vascular inflammation nor atherosclerotic lesion size.

A small but significant increase in CD68+ macrophage staining was observed in *LdlrKO* mice transplanted with *GPR35KO* bone marrows. If not for the fact that the GPR35KO group presented no difference in other inflammatory markers evaluated and atherosclerosis, increased macrophage infiltration would be expected in this group based on previous data indicating that ligation of GPR35 can reduce monocyte/macrophage activation, for example KynA decreased the LPS-induced TNF secretion [5]. However, in our study, treatment with a more potent and specific GPR35 agonist than KynA, zaprinast, did not influence macrophage activation in response to LPS. While this seemed contradictory at first, it should be remembered that KynA can also exert anti-inflammatory activity via signaling through the aryl hydrocarbon receptor (AhR) and the α7 nicotinic acetylcholine receptor [17]. In fact, during the development of the present project, we showed that KynA-mediated signaling through AhR, and not the GPR35, regulated macrophage activation and the secretion of TNF and IL-6 [18]. Altogether, our data suggest that GPR35 does not influence the activation of immune cells, more specifically myeloid-derived cells, and that its potential role in inflammation could be associated with its expression in other accessory cells to the immune system or other tissues. The fact that BMDMs from GPR35KO and WT do not differ in their response to LPS corroborates with this notion.

In conclusion, we have demonstrated that the lack of GPR35 expression in blood cells does not influence vascular inflammation and atherosclerosis. Nevertheless, considering that atherogenesis is influenced by several risk factors, including comorbidities like obesity and other auto-immune diseases that have been associated with GPR35, our study does not completely rule out its potential relevance for CVDs. Future studies targeting other relevant cells or tissues in the process of atherosclerosis, for example vascular endothelial cells and smooth muscles cells will be necessary to reach a more definitive conclusion.

## 4. Materials and Methods

### 4.1. Animals and Bone Marrow Transplantation

Heterozygous cryopreserved sperm from C57BL/6 mice with a targeted deletion of *Gpr35* (Gpr35^tm1(KOMP)VIcg/MbpMmucd^), obtained from the KOMP Repository Project (University of California, Davis, USA; https://www.komp.org/ access on 23 June 2021), was used for in vitro fertilization, and heterozygous breeding of animals was used to generate GPR35KO and littermate wild type (WT controls). Detailed information on mice genotyping is presented in Supplemental materials (Figure S3, and Supplemental Methods). Real-time PCR analysis of organs confirmed the complete ablation of *Gpr35* expression in KO mice (Figure S4).

In-house bred low-density lipoprotein receptor KO mice (*LdlrKO*) expressing the CD45.1 allele on a C57BL/6 background (as described in Klingenberg et al. [19]) were used as recipients in bone marrow transplantation experiments. In brief, bone marrow cells isolated from the femur and tibia of male *GPR35KO* or WT mice (both expressing the CD45.2 allele) were intravenously injected (5 × 10^6^ cells per mouse) into lethally irradiated (7 Gy; two times 3.5 Gy, 3 h apart) 13 week-old male and female *LdlrKO* mice. Antibiotics (Tribrissen) were administered for 3 weeks to all mice. Four weeks after the irradiation, the chimeric mice were fed a western diet [corn starch, cocoa butter, casein, glucose, sucrose, cellulose flour, minerals, and vitamins comprising 17.2% protein, 21% fat (62.9% saturated, 33.9 unsaturated, and 3.4% polyunsaturated), 0.15% cholesterol, 43% carbohydrates, 10% H_2_O, and 3.9% cellulose fibers; R638 Lantmännen, Sweden] ad libitum, for 8 weeks. Efficiency of bone marrow chimerism is shown in Figure 1A. All animal experiments were performed in accordance with national guidelines and approved by the Danish Ministry of Food, Agriculture and Fisheries and the Stockholm Norra regional ethics board, which conform to the guidelines from Directive 2010/63/EU of the European Parliament on the protection of animals used for scientific purposes.

### 4.2. Tissue Collection, Lesion Analysis, and Immunohistochemistry

At the end of WD treatment, mice were euthanized with CO_2_. Blood was collected by cardiac puncture, and vascular perfusion was performed with sterile RNase-free PBS. After perfusion, heart and the aortic arch were dissected and preserved for lesion and immunohistochemistry analyses. *En face* lipid accumulation was measured in the aortic arch using Sudan IV staining. Plaque area was calculated as the sum of total plaque area in the aortic arch (excluding branching vessels). Aortic root lesions were evaluated in hearts from male transplanted mice that were serially sectioned from the proximal 1 mm of the aortic root on a cryostat. Lesion size was determined from 8 hematoxylin- and Oil Red O-stained sections, collected every 100 μm over a 1 mm segment of the aortic root. Lesions were quantified as previously described [20].

Primary rat anti-mouse antibodies to CD68 (AbD Serotec, Oxford, UK), CD3 (Southern Biotech, Birmingham, AL, USA), VCAM-1 (BD Biosciences, Franklin Lakes, NJ, USA), and rabbit anti-mouse antibody to alpha smooth muscle cell actin (Abcam, Cambridge, UK) were applied to acetone-fixed cryosections, followed by biotinylated rabbit anti-rat or goat anti-rabbit IgG. Mouse anti-mouse IA^b^ (BD Biosciences, Franklin Lakes, NJ, USA) was conjugated directly to biotin. Staining reactions developed using the VECTASTAIN ABC kit and diaminobenzidine (Vector Laboratories, Burlingame, CA, USA). Immunohistochemical data was obtained using Qwin computerized analysis (Leica, Wetzlar, Germany) of stained sections. Samples that were compromised during processing or analysis were excluded from the study. For the assessment of plaques, samples were coded, and the evaluation performed by trained persons, which were blinded to the treatment groups.

### 4.3. Bone Marrow-Derived Macrophage (BMDM) Experiments

Bone marrow cells were extracted from the femurs and tibias of GPR35KO and WT littermate controls, differentiated in medium (RPMI, 10% FBS, 200 IE/mL penicillin, 50 μg/mL streptomycin, 1 mmol/L sodium pyruvate, 2 mmol/L l-glutamine) supplemented with 100 ng/mL mMCSF (Peprotech, Rocky Hill, NJ, USA) for 7 days as previously described [21] and then counted and plated for experiments. To measure BMDM responses, cells were plated at 1 × 10^6^ cells/mL and stimulated with different concentrations of LPS (Enzo, Farmingdale, NY, USA) for 2 and 20 h. The GPR35 strong agonist, zaprinast (Sigma, St. Louis, MO, USA), was used in the presence of 0.1 ng/mL LPS to investigate the effects of the receptor on macrophage activation. At the end of the incubation times, cells and supernatants were harvested for mRNA gene analysis using qPCR, and the detection of cytokines by MSD, respectively.

### 4.4. mRNA Isolation and Analysis

RNA was isolated from mouse organs using the RNeasy Lipid Tissue Mini kit (Qiagen, Hilden, Germany) according to the manufacturer’s instructions. RNA was isolated from BMDMs using the NucleoSpin RNA kit (Macherey Nagel, Düren, Germany) according to the manufacturer’s instructions. After measuring mRNA concentration on a Nanodrop 2000 Spectrophotometer (Thermo Scientific, Waltham, MA, USA), cDNA was synthesized using the SuperScript III kit (Thermo Fisher, Waltham, MA, USA). Quantitative gene expression analyses were performed using PrecisionPLUS qPCR MasterMix with SYBRgreen (Primer Design, Camberley, UK) and validated primers (Table S2) on a LightCycler^®^ 480 II (Roche, Basel, Switzerland) thermocycler. Tyrosine 3-monooxygenase/tryptophan 5-monooxygenase activation protein zeta (*Ywhaz*) was used as housekeeping gene. Data were analyzed based on the relative expression method with the formula 2^−ΔΔCT^, where ΔΔCT = ΔCT (sample) − ΔCT (calibrator = average CT values of all samples within each group) and ΔCT is the average CT of the housekeeping genes subtracted from the CT of the target gene.

### 4.5. Cytokine Analysis

Cytokines in the supernatant to activated BMDMs, IL-1β, IL-6, IL-10, CXCL1, and TNF (V-PLEX^®^ Proinflammatory Panel 1 Mouse kit), were assessed using MSD (Mesoscale Diagnostics, Rockville, MD, USA) according to the manufacturer’s instructions. The plates were analyzed on a MESO QuickPlex SQ 120 (Mesoscale Diagnostics, Rockville, MD, USA) using the plate barcode, and data was analyzed using the MSD Discovery Workbench software version 4.0.12 (Mesoscale Diagnostics, Rockville, MD, USA).

### 4.6. Statistical Analysis

The results are presented as the mean ± SEM if not otherwise stated. The Mann–Whitney U-test was used for comparison between two groups. Two-way ANOVA was used to estimate quantitative changes between the groups. Friedman test with Dunn’s post-test was used for pairwise comparisons between groups in the cell experiments. *p*-values < 0.05 were considered significant.

## Data Availability

The data presented in this study are available on request from the corresponding author.

## Supplementary Materials

The following are available online at https://www.mdpi.com/article/10.3390/metabo11070411/s1, Table S1: Primer sequences for GPR35KO and WT genotyping by PCR setup, Table S2: qPCR primer sequences, Figure S1: Representative gels of GPR35KO and WT mice genotyping, Figure S2: Relative expression of *Gpr35* in different organs of WT and KO mice, Figure S3: Dose titration of GPR35 agonists, Figure S4: The effect of GPR35 ligation with zaprinast to CXCL1 and IL-10 production on BMDMs stimulated with LPS.
