# Supplementary material for: Disruption of GPR35 Signaling in Bone Marrow-Derived Cells Does Not Influence Vascular Inflammation and Atherosclerosis in Hyperlipidemic Mice
Source: Metabolites. 2021 Jun 23;11(7):411. doi: 10.3390/metabo11070411 (PMC8303390; doi:10.3390/metabo11070411)
Supplement: Supplementary file 1 [file metabolites-11-00411-s001.zip › metabolites-1264532-supplementary.pdf]

Disruption of GPR35 Signaling in Bone Marrow-Derived Cells Does Not Influence Vascular Inflammation and Atherosclerosis in Hyperlipidemic Mice

Roland Baumgartner[1], Felipe B. Casagrande[1], Randi B. Mikkelsen[2], Martin Berg[1], Konstantinos A. Polyzos[1], Maria J. Forteza[1], Aastha Arora[1], Thue W. Schwartz[2], Siv A. Hjorth[2] and Daniel F. J. Ketelhuth[1,3,*]

1 Division of Cardiovascular Medicine, Center for Molecular Medicine, Department of Medicine, Ka-rolinska Institute, Karolinska University Hospital, Stockholm, Sweden; roland.baumgartner@ki.se (R.B.); felipe.casagrande@ki.se (F.B.C.); clmbbbk@gmail.com (M.B.M.); konstantin.polyzos@gmail.com (K.A.P.); maria.forteza.de.los.reyes@ki.se (M.J.F.); aastha.arora@ki.se (A.A.)

2 Novo Nordisk Foundation Center for Basic Metabolic Research, University of Copenhagen, Copenhagen, Denmark; mikkelsen@sund.ku.dk (R.B.M.); tws@sund.ku.dk (T.W.S.); sivhjorth@sund.ku.dk (S.A.H.)

3 Department of Cardiovascular and Renal Research, University of Southern Denmark, J.B. Winsløws vej 21, 3, 5000 Odense C, Denmark

* Correspondence: ketelhuth@health.sdu.dk

SUPPLEMENTAL MATERIAL

SUPPLEMENTAL METHODS

GPR35KO genotyping

The mice were genotyped using DNA from tail snips. The tail snips were lysed and subsequently neutralized before being used in the PCR on an Eppendorf Mastercycler®. The PCR products were analyzed by 2% agarose gel electrophoresis. Two primer sets were used to detect the KO and WT alleles, respectively (Supplemental Table 1).

Evaluation of bone marrow transplantation efficiency by flow cytometry

Flow cytometry was performed on erythrolysed blood from transplanted mice at the end of the experiment. Primary labeled antibodies were used for extracellular staining against CD45 isoforms. The cells were incubated for 15 min at room temperature with FcBlock (anti–CD16/CD32; eBioscience, CA, USA). Subsequently, the cells were washed and incubated for 30 min at 4°C with CD45.1-eFluor 450 and CD45.2-APC-eFluor 780 (both from eBioscience, CA, USA). The samples were washed, fixed in 1% formalin, and analyzed using a Dako CyAn (Beckman Coulter, CA, USA) flow cytometer and FlowJo 10.3 software (Treestar).

SUPPLEMENTAL TABLES

Table S1. Primer sequences for GPR35KO and WT genotyping by PCR setup.

Primer	Sequence (5'-3')	WT	KO
1) Reg-NeoF	GCAGCCTCTGTTCCACATACACTTCA	0	546
2) Reg-Gpr35-R	AAGAAGCCATTTGAGGGTCCTCTGG		
3) Reg-Gpr35-wtF	CTCCTTGCCATTTGTGCTGTACTCC	339	0
4) Reg-Gpr35-wtR	AGGTAGAATCCCAGCAGTGAGAAGG		

Table S2. qPCR primer sequences

Gene	Forward (5'-3')	Reverse (5'-3')	Amplicon size, bp
Ywhaz	AGACGGAAGGTGCTGAGAAA	GAAGCATTGGGGATCAAGAA	127
Gpr35	GCCTGGATGCCATCTGTTAC	TCAGGATCTGGGAATCTTGG	108
Tnf	GTAGCCCACGTCGTAGCAAA	TTGAGATCCATGCCGTTGGC	95
Il1b	GCCACCTTTTGACAGTGATGAG	GACAGCCCAGGTCAAAGGTT	95
Il6	AGTCCTTCCTACCCCAATTTCC	TGGTCTTGGTCCTTAGCCAC	80
Il10	GCATGGCCCAGAAATCAAGG	GAGAAATCGATGACAGCGCC	91
Cxcl1	ACTCAAGAATGGTCGCGAGG	GTGCCATCAGAGCAGTCTGT	123

SUPPLEMENTAL RESULTS

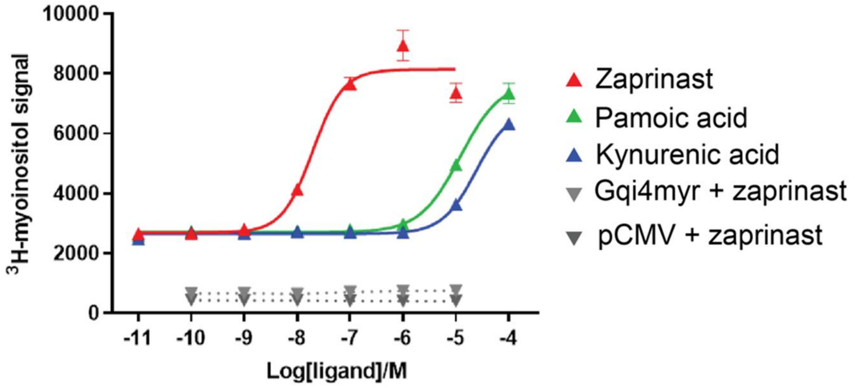

Figure S1. Dose titration of GPR35 agonists. IP3 production was evaluated in COS cells transfected with mouse GPR35, or modified G protein Gqi4myr or pCMV as controls was conducted as previously described [1]. Cells were stimulated with three previously described agonists, kynurenic acid, pamoic acid, and zaprinast, at increasing concentrations. The assay shows that zaprinast is the most potent agonist among the three tested.

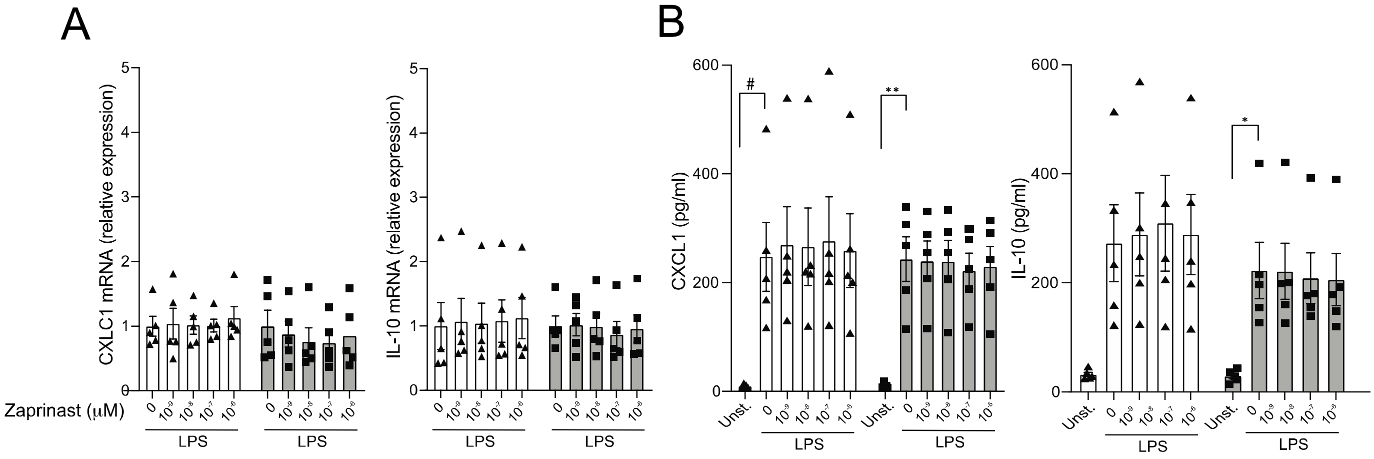

Figure S2. The effect of GPR35 ligation with zaprinast to CXCL1 and IL-10 production on BMDMs stimulated with LPS. A) Relative mRNA expression of *Cxcl1* and *Il-10* in GPR35KO and WT mice, co-incubated for 2 h with increasing doses of zaprinast and 0.1 ng/ml LPS; n = cells from 5 mice/ group. B) Levels of CXCL10 and IL-10 secreted by BMDMs from GPR35KO and WT mice, co-incubated for 2 h with increasing doses of zaprinast and 0.1 ng/ml LPS; n = cells from 5 mice/ group. Results are mean ± SEM. No significant differences were observed; Friedman test with Dunn's

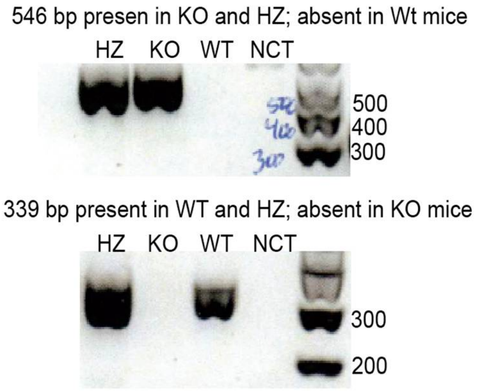

Figure S3. Representative gels of GPR35KO and WT mice genotyping. Representative pictures from agarose gel electrophoresis of GPR35 genotyping sequences amplified by PCR. From left to right it shows heterozygous (HZ), homozygous knockout (KO), homozygous wildtype (WT), no template control (NCT), and ladder control (base pairs size). Top gel shows the result of PCR to detect the mutant allele using primers #1 and #2 (Supplemental table 1). Bottom gel shows the result of PCR to detect the wildtype allele using primers #3 and #4 in (Supplemental table 1).

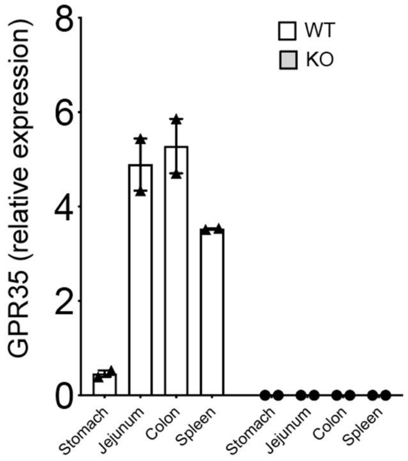

Figure S4. Relative expression of *Gpr35* in different organs of WT and KO mice. *Gpr35* mRNA expression quantified by qPCR in stomach, jejunum, colon and spleen of GPR35KO and WT littermate controls (n = 2). Results are mean ± SEM. *Gpr35* mRNA was undetectable on organs from GPR35KO animals.

SUPPLEMENTAL REFERENCES

1. Trauelsen, M., et al., *Receptor structure-based discovery of non-metabolite agonists for the succinate receptor GPR91.* Mol Metab, 2017. **6**(12): p. 1585-1596.
